# The vigilance decrement: its first 75 years

**DOI:** 10.3389/fcogn.2025.1632885

**Published:** 2025-11-24

**Authors:** Raymond M. Klein, Brett B. T. Feltmate

**Affiliations:** Department of Psychology and Neuroscience, Dalhousie University, Halifax, NS, Canada

**Keywords:** criterion, sensitivity, signal detection theory, sustained attention, watch keeping

## Abstract

The first major laboratory studies of vigilance by Mackworth in 1948 and later revealed a decline in the probability of detecting brief targets as the time on task increases. Whether referred to as a vigilance decrement or something else (e.g., a failure of sustained attention), because such failures have great applied significance (e.g., in road safety, radiology, air-traffic control, civil defense, etc.), understanding the vigilance decrement and discovering ways to avoid it are important goals for psychological science. The purpose of this historical review is to provide a picture of the extensive scientific literature exploring the nature(s) of the vigilance decrement, with an emphasis, but not exclusionary focus, on the signal detection theory framework. Beginning in the early 1960s, researchers started to interpret this decline in target detections using signal detection theory, wherein a decrease in detections can be attributed to a decrease in sensitivity of the observer to the difference between targets and non-targets, a conservative shift in the observer's response criterion, or, of course, both. Some early investigators suggested that which of these two causes of the decline in detections is operating may depend on the rate at which events (targets and non-targets combined) are presented: When the event rate is slow, criterion shifts dominate detection failures, whereas declines in sensitivity become more pronounced as event rates increase. Nevertheless, the contribution of sensitivity declines has been recently challenged. One source of the challenge is the relatively low false-alarm rate in so many studies on the vigilance decrement. Another is the possibility that for a variety of reasons, the observer in a relatively long vigil may stop attending to the source of the task-relevant signals. Some recommendations are offered based on our reading of the ~75 years of vigilance research.

## 1 Introduction

It has recently been asserted (Klösch et al., 2022) that the definition of *vigilance* is “unclear and ambiguous.” We disagree. Among scholars interested in human perception and performance, there is little disagreement about what *vigilance* means, and that meaning is well-captured by the dictionary (Merriam Webster) definition: “the state of being … alertly watchful especially to avoid danger.” If there is any confusion among neurologists, this may be because of an overemphasis attributed by Klösch et al. to Head (1923) on the metaphorical application of the everyday term when referring to *vigilance* “as the organism's ability to reorganize itself and restore damaged functions.” That noted, we agree with the subtitle of Head's (1923) article that vigilance is “a physiological state of the nervous system” and with the assertion that “vigilance is a universal property of animals and humans in order to react adequately to environmental stimuli and to ensure the survival of the individual” (Klösch et al., 2022, p. 2). Whereas, there is good agreement that vigilance is a neuropsychological (in the sense meant by Hebb (1949), viz. neural *and* psychological) state of the organism that entails being “alertly watchful,” there is no good agreement about how this state, or changes in it, affects our ability to detect and respond appropriately to signals whether dangerous or not. Our purpose is to provide a picture of the extensive scientific literature exploring the nature or natures of the vigilance decrement: a historical review with an emphasis, but not exclusionary focus, on the signal detection theory framework.

By “changes in vigilance,” we refer primarily to the ubiquitous decline in the detection of and response to occasional targets presented during relatively long vigils: the aptly named “vigilance decrement.” The beginning of the experimental analysis of the vigilance decrement is typically attributed to Mackworth's (1948) article titled “The Breakdown of Vigilance During Prolonged Visual Search” and his later monograph (see also Mackworth, 1961), wherein a decrement in performance with both visual and auditory targets was shown to be robust after the first half hour on the task. Importantly, in this monograph, Mackworth discovered that a salient interruption (telephone call or a half-hour break from the task) or the provision of performance feedback could reduce or eliminate the decrement while urging the subject to be especially attentive had no effect. Whereas, quite a few theories have been advanced to explain what causes the vigilance decrement, our focus in this review is on describing the changing views about the nature of the decrement, that is, what its effects are, and trying to reach a firm conclusion about the nature of these effects.

## 2 From 1948 (N. Mackworth) - until 1963

**Figure 1:**
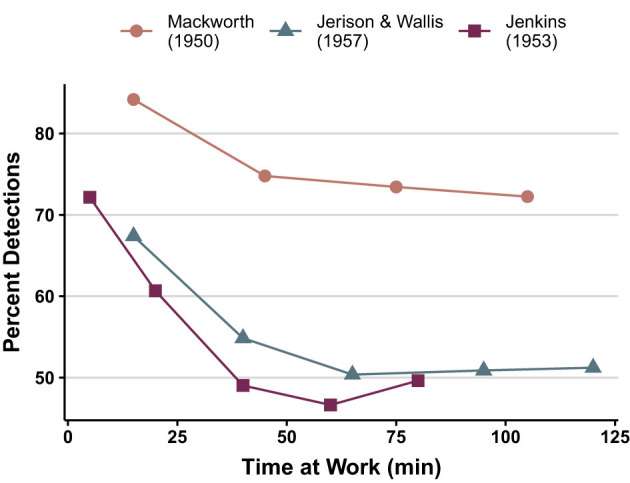
Three performance curves from different laboratories illustrating the decrement finction in human vigilance. All curves based on signal rates of about 30/hour and display with no spatial uncertainty (redrawn from Jerison and Pickett's, 1963, Figure 1).

Between the empirical discovery of the vigilance decrement and 1963, the decrement was aptly described and operationalized as an increase in the failure to detect targets with time on task (see Figure 1). A quick survey of this early literature indicates an interest in how various stimulus factors (signal rate, duration, and intensity), situational factors (multitasking, visual deprivation, noise, interpolated rest, sensory modality, drugs, knowledge of results, distracting [non-task-related] events), and participant attributes (authoritarianism, aging, and extraversion/introversion) might affect vigilance and its decrement with time on task. It is noteworthy that these interests have continued unabated to this day, with an increasing emphasis on sleep deprivation and the collection of neuroscientific data.

Whereas, most researchers have focused on the probability of reporting the presence of a target, from the very beginning (e.g., Mackworth, 1950), the reaction times (RTs) of detection responses were examined by a handful of scholars. McCormack (1958), for example, reported that detection response times increased during the course of a 40–50-min vigil and that an interruption after 40 min generated an improvement (reduction) in RT that was greater with a longer interruption. Jenkins (1958) used a complex dual task wherein the participant made button-press responses to occasional light flashes while also monitoring a clocklike device for double-step (the eponymous NH Mackworth Clock) targets. In a group with no interruptions, he found that target detection decreased while RTs to light flashes increased during the course of a vigil. In a group with regular (every 5 min) interruptions (30-s duration), both of these effects were markedly reduced. Examining the relationship among this study's dependent variables led Jenkins (1958) to this interesting finding:

Taken together, [these data] show that, on the average, when S was responding rapidly to the light-signal he was at his best both in detecting pointer-signals and in avoiding false reports. (p. 656)

One obvious inference is that the mental state of being alert is characterized by faster RTs and better detection performance. From a review of the early literature on this topic, Buck (1966) concluded that the evidence “supports the assumption that reaction-time increment and detection-rate decrement are indices of change of the same psychological variable” (p. 302).

In 1961, the U.S. Office of Naval Research funded a symposium on the topic of vigilance. The proceedings were later published (Buckner and McGrath, 1963) not only with the presented papers but also with most of the discussion following each one. Most important, from this review's point of view, is the “lesson” provided by Broadbent (1963) in the discussion that followed his first presentation. In this presentation, Broadbent refers to an experiment:

[By] myself and Margaret Gregory. In it the observation by Colquhoun that in some of his studies the rate of false detections (reports of non-existent signals) change in step with the rate of detections, and upon similar results from the Royal Navy. Now most studies of vigilance pay relatively little attention to false detections. The reason for this, at least as far as I am concerned, has been an implicit belief in the applicability of the “guessing correction” for such errors.”

(p. 79)

The guessing correction assumes that on some percent of trials without a signal, the observer may guess; the rate of such guesses can be determined by the rate of false detections, and this can be used to correct (lower) the rate of correct detections to account for the accidentally correct guesses. The “guessing” explanation for false detections was then contrasted with the signal detection theory (SDT) advanced by Tanner and Swets (1954), and the aforementioned experiment utilized confidence ratings as proposed by Egan et al. (1959). According to Broadbent, the data were inconsistent with the guessing model and consistent with the idea that the “deterioration during a watch period may be the result of a shift in criterion level” (p. 81).

**Figure 2:**
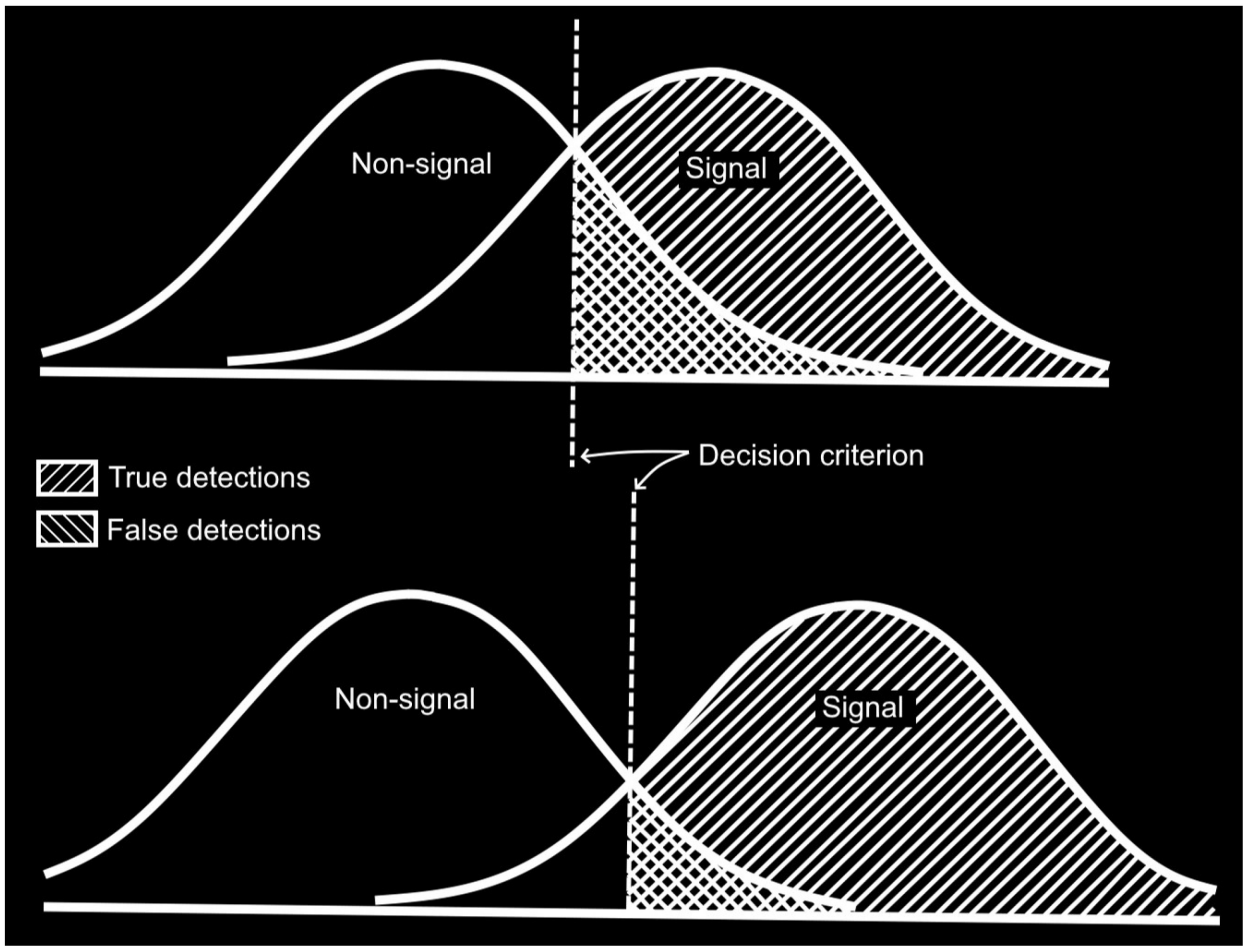
Idealized representation of Broadbent's illustration of the basic ideas in signal detection theory as drawn on a blackboard during the discussion of his presentation at *Vigilance: A Symposium* in 1961 (from discussion of Broadbent, 1963).

During the discussion (Buckner and McGrath, 1963, pp. 84–85), one of the symposium participants reported that practice (from 1 day to the next) resulted in a decrease in false detections and an increase in correct detections, saying: “I suppose you would say that the observer's criteria for signals had become more stringent with practice,” to which Broadbent responded, “No, that is not so.” He went on to use the blackboard to explain the fundamental ideas behind SDT. The effect of practice described by the participant would be explained by an increase in the separation of the signal and non-signal distributions (as illustrated in Figure 2). This can increase the rate of true detections (hereafter, “hits”) while decreasing the rate of false detections (hereafter, “false alarms” or FAs). Later in the discussion, Broadbent explained:

I am interested in what explains the downward trends in performance during a watch, and why performance recovers after a rest pause, and why it changes between alerted and non-alerted conditions. Now these performance changes and others like them I strongly suspect are not due to changes in *d*′, but are due to changes in subjects' decision criteria.

(Broadbent, 1963, p. 86)

And he went on to explain how the two SDT parameters are defined: d′ (or sensitivity) as the distance between the distributions divided by their standard deviations and beta (or the criterion) as the ratio of hits to false alarms.

In this period a variety of theories were proposed to explain vigilance and its decrement with time on task. As reviewed by Frankmann and Adams (1962), the foundations for some of these were rooted in the then still-dominant animal learning literature, such as Pavlovian inhibition, extinction, and habituation. Others, such as Deese's (1955) emphasis on expectancy and Broadbent's (1953) emphasis on attention and distraction, might be considered at the cusp of the cognitive revolution. Hebb's (1955) ideas about motivation, the importance of stimulus variety, and the role of the reticular activating system for maintaining alertness and arousal might be considered to play a role regardless of one's theory. At this point, there is little reason to delve into these theories in detail (but see Section 8.1).

First, as Frankmann and Adams (1962) summarize them at the beginning of their concluding section, these early theories were not presented precisely enough:

The main shortcoming of our contemporary theories of vigilance would seem to be a casualness of formulation that makes the definitive testing of implications rather difficult.

(p. 268)

Whereas, we agree with the implication here that theories ought to be computationally explicit, frankly, and perhaps iconoclastically, we wonder if the effort to prove one's theory was (and perhaps still is) a contributor to a lack of progress. As Broadbent (1958, p. 307+) pointed out, we do not learn much from the typical hypothetico-deductive experiment organized around the logic: “If our theory is correct, and we manipulate X then we should find Y.” But from a thorough consideration of all the empirical data (without theoretical “blinders”), it may be possible to derive some solid inferences that could provide the basis for a sound explanation or, more likely, a set of them.

Second, as anticipated in Broadbent's 1961 (1963) symposium presentation, beginning around 1963, many researchers began incorporating SDT into their understanding of target detection performance in vigilance experiments. This conceptual and methodological advance allowed the decrease in the detection of targets (illustrated in Figure 1) to be explained in two ways: a decrease in the observer's sensitivity to the difference between targets and non-targets (or noise) or a conservative shift in the observer's decision criterion for indicating that a target had been detected (or, of course, both). It is worth noting, for readers interested in the RT to targets, that either of these effects ought to increase RT.

## 3 1963–1969

Three papers were published in 1963 that explicitly recognized the value of applying SDT for understanding the nature of vigilance decrements. A review paper by Jerison and Pickett (1963) provides a thorough review of the literature up until then. Perhaps stimulated by Broadbent's aforementioned symposium presentation, they suggest that

[o]ne approach has been to apply signal detection theory directly to the vigilance problem (Broadbent, 1963), and to treat the decrement function, for example, as the result of changes during a vigil in the observer's criterion for deciding that a particular event was “signal” or “noise.”

(p. 212)

The work by Broadbent and Gregory (1963) (that was alluded to in Broadbent's 1961 presentation (Broadbent, 1963) and referred to here by Jerison and Pickett) was published in 1963 with the title: “Vigilance considered as a statistical decision.” Before presenting their experiments, SDT is explained, and it is noted that “[a] drop in detections alone can be due to a decrease in d′ with a constant beta, or a rise in beta with a constant d′” (p. 313). In experiments focused on the effects of noise and time on task with auditory and visual targets, it was concluded that the effect of time on task was a conservative shift in the decision criterion. Despite this finding, it is ironic that the telltale, if not definitional, characteristic of a vigilance decrement (a decline in target detections) was not observed in this study.

In the same year, Mackworth and Taylor (1963) began their paper, stating:

In a vigilance task, the index of performance is usually the percentage of signals correctly reported. Many studies, however, whose main points have been set out by Swets et al. (1961), have demonstrated that this index is not a suitable measure of the detection efficiency of the observer.

(p. 302)

Using several versions of the continuous-clock task (wherein the signal is a brief pause in the otherwise steady movement of a clock hand), with most conditions having a substantial level of false alarms, they concluded that

there is a reduction in the detectability of a signal, measured by d′, as time on watch proceeds, even when the subject knows the distribution of the signals and when the criterion for response is irrelevant, as with forced choice.

(p. 324)

Thus, in two of the earliest published empirical papers that applied SDT to the vigilance decrement, there was immediately a dramatic conflict, with Broadbent and Gregory attributing the decline in hits to a more conservative criterion and J. Mackworth and Taylor attributing it to a decline in sensitivity. We will revisit this discrepancy shortly and somewhat regularly in this review.

But another possible cause of the decline in hits requires our attention. It was clearly alluded to by Jerison and Pickett (1963) immediately after they first mentioned SDT (in the previous quote): “We have been following a somewhat different approach at this laboratory by emphasizing the observer's decisions about whether or not to attend to the display” (p. 212).

The concept of attention had been relegated to “mentalistic” status by behaviorists in the first half of the 20th century and, around 1963, was (principally because of Broadbent's (1958), seminal book) only just beginning to return to scientific respectability. Whether or not their use of it here is considered courageous, it certainly represents an important, if obvious, third possible cause of the decline in detections with time on task. Jerison and Picket link their use of attention to the idea of an “observing response” from a methodological feature in some animal learning experiments (wherein the animal must make a response to produce the events of a trial) while also distinguishing it from this operant methodology. It is clear from their description that *orienting* is a more appropriate term. For visual targets, they describe this as a chain of events that begins with “neuro-muscular orientations: pointing the head and eyes, convergence of the two eyes, and accommodation of the optic lens system,” which allows the stimulus to generate activity in the visual pathway. In today's Posnerian terminology, this would be referred to as overt orienting. But despite using the idea “decision to attend,” they later refer to “these orientations” as “voluntary or involuntary,” thus allowing for endogenous and/or exogenous overt orienting to play a role in the vigilance decrement with visual stimuli. Later, they point out that decrements in vigilance can be observed in modalities, such as audition and touch, for which the aforementioned overt orientation adjustments do not occur. We see this as allowing for covert orienting (attention) or distraction (as described by Jerison et al., 1965) to play a role. Without using contemporary terminology, Jerison and Pickett were thus allowing for the endogenous and/or exogenous control of covert and/or overt orienting to play a role in the relatively ubiquitous vigilance decrement; that is, not attending the source (modality or location) of important signals whether through overt or covert means (such as falling asleep, looking away or thinking about something else as in daydreaming or mind wandering) would result in a period of fewer responses (fewer hits and fewer false alarms). This is critically important because none of these orienting adjustments is the same as a more conservative decision criterion or as a decline in sensitivity. Yet, in the context of SDT, the absence of responses (both hits and false alarms) during a portion of a watch would be difficult to distinguish from a conservative shift in the decision criterion combined with a decline in sensitivity.

Let us return to the aforementioned discrepancy. Aside from the difference in the nature of the vigilance tasks used in these two studies, another potentially important difference might be the rates of responding: The rate of responding was much higher in Mackworth and Taylor (every 5 s) relative to Broadbent and Gregory (averaging about one or fewer responses every minute).

In 1965, Broadbent and Gregory examined the vigilance decrement in experiments in which there were regular visual stimuli (every 3.5 s), the target was a slightly brighter flash whose frequency varied between experiments (from ~ 3.5–1 per minute), and on each stimulus presentation, participants had to make four choice responses (yes/no with two levels of confidence, sure/not sure). In agreement with their earlier findings, they concluded that “[i]t will be seen that there is no indication whatever of a fall in d′ as a watch in quiet proceeds” (p. 159). This conclusion is contrasted with Mackworth and Taylor's findings of consistent declines in d′, and most of the discussion of the paper is aimed at trying to explain this discrepancy. In the end, it is concluded that

[a]t the present time therefore the least improbable interpretation of the difference between the two sets of results is that situations involving difficult sensory adjustments may actually show a drop in the sensitivity of the operator during the watch, while others will merely show an increased reluctance to report signals of which he is doubtful.

(Broadbent and Gregory, 1965, p. 162)

Frankly, this “least improbable” explanation of this early discrepancy is highly unlikely.^1^ Mackworth and Taylor (1963) observed a vigilance decrement in d′ regardless of the overall difficulty of the task (with d′s ranging from 3.0 to 1.0), and Broadbent and Gregory's (1963, 1965) discrimination difficulties were right in the middle of this range. This point aside, the conflict remains: Mackworth and Taylor (1963) obtained a robust decline in d′ during their watches, while Broadbent and Gregory (1965) did not.

**Figure 3:**
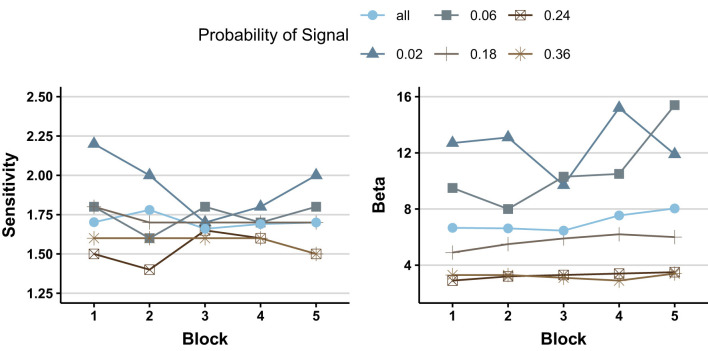
Sensitivity (d′) and criterion (β) as a function of signal probability and (block) time on task (graphic illustration of the data from Table 4 of Baddeley and Colquhoun, 1969).

This period is nicely capped off by Baddeley and Colquhoun's (1969) exploration of the effect of signal probability upon performance as a function of time on task. On each trial, one every 2 s, a row of 6 disks was presented. The target was when one of the disks was a little larger than the others, and the probability of target trials varied between 2% and 36%. The authors were primarily interested in how signal probability would affect hits and false alarms, both of which increased with increases in signal probability. For present purposes, we are primarily interested in how time on task affected performance. After showing that both hits and false alarms tended to decrease with time on task (more so in some conditions than others), they shifted their attention to the aforementioned measures from SDT. As can be seen in Figure 3, β computed as C here, was markedly affected by signal probability, with a shift from liberal to conservative as signal probability increased, and it increased significantly (although not a lot) with time on task (more so with lower signal probabilities). Collapsed across signal probabilities, and in line with the two Broadbent and Gregory findings described earlier, d′ was unaffected by time on task.

## 4 1970–1980

Several summaries of the literature on vigilance decrements, with a focus on whether and, if so, when the decline in hits would be associated with a decline in sensitivity were published in this decade.^2^

The first of these was embedded in an *Annual Review of Psychology* article on attention by Swets and Kristofferson (1970). Because Swets is one of the developers of SDT, how he critically examined the literature is valuable for this review. While most of this review is focused on topics stimulated by Broadbent's (1958) influential theory of selective attention, the last section is about SDT approaches to vigilance and the vigilance decrement. This section on vigilance is organized into several subsections.

The first section, titled “Is There a Sensitivity Decrement? No,” begins with the two studies by Broadbent and Gregory covered earlier. The conclusions of these two papers, that there was a conservative shift but not a decrease in d′ during a watch, were accepted (although the aforementioned absence of a decline in hits in the 1963 paper was raised as a caution), Importantly, in the remainder of this section, more than 10 studies that found no decrement in d′ are reviewed. The next section, titled “Is There a Sensitivity Decrement? Yes,” begins with the aforementioned study by Mackworth and Taylor (1963) and then notes that using the same continuous-clock test, a similar decline in d′ was observed in five different studies by one or the other of these two authors. The last section of this empirical review, titled “Is There a Sensitivity Decrement? Yes and No,” begins with two studies by J. Mackworth that were aimed at the aforementioned discrepancy. One of these (Mackworth, 1965), using occasionally brighter flashes as targets, found a decrement in d′ when the background rate of non-signals was high but not when this rate was slower. In a later experiment, Mackworth (1968a) compared the continuous-clock task (used in Mackworth and Taylor, 1963) with the jump-clock task used by N. H. Mackworth. A decrement in d′ was only seen with the continuous-clock task. Three other studies were mentioned in this section.

Swets and Kristofferson (1970) then offer suggestions for future research on the vigilance decrement when aimed at obtaining measures from SDT:

1. Modify procedures of vigilance experiment to be more like those of traditional psychophysical experiments.
2. Use weaker signals so sensitivity, d′, is between 1 and 2.
3. Aim for higher FA rates (e.g., >2%) so that its estimation may be more reliable.
4. Try to obtain receiver operating characteristic (ROC) curves.^3^

After this review with its explicitly eclectic pattern of results, the authors conclude that

[i]t is clearly important to continue trying to spell out what kinds of displays yield reduced sensitivity over time. and what kinds of displays do not suffer with passing time.

(Swets and Kristofferson, 1970, p. 362)

This advice would later provide the rationale for Parasuraman's (Parasuraman and Davies, 1977; Parasuraman, 1979) seminal taxonomy and See et al's (1995) meta-analysis.

In Broadbent (1971) second magnus opus, *Decision and Stress*, two early chapters were devoted to vigilance, both with a focus on theory: The first covers traditional measures while the second focuses on studies rooted in SDT. The conclusions from the second of these two chapters are particularly pertinent. From an empirical point of view, Broadbent suggests that

a. with high event-rate tasks (including those with transient signals occurring at unexpected times), a decrement in d′ is expected with time on task;
b. with transient signals and a low event rate, there is no decrement in d′, but there is an increasingly conservative criterion for responding with time on task; and
c. with non-transient signals, there is no decrement in detections.

From the various failures of the extant theories to explain one finding or another, Broadbent (in a reprise from his 1958 book) concluded that “[i]t has long been clear that no single theory would encompass all of the phenomena of vigilance” (p. 109).

In a chapter “The Psychobiology of Attention,” published in Blakemore and Gazzaniga's *Handbook of Psychobiology*, Posner (1975) describes a field in which behavioral and neuroscientific approaches to attention are isolated and suggests that it would be fruitful to integrate the two approaches. He distinguishes three components of attention, which he refers to as not mutually exclusive, and interrelated: alertness, selectivity, and conscious effort.

Posner defines alertness as the state of the organism that affects general receptivity to stimuli, and he distinguishes phasic (fast, abrupt) changes in alertness associated with warning signals from tonic (slow, gradual) changes in alertness, such as those associated with the circadian sleep-wakefulness rhythm and long periods of continued performance, namely, vigilance.

**Figure 4:**
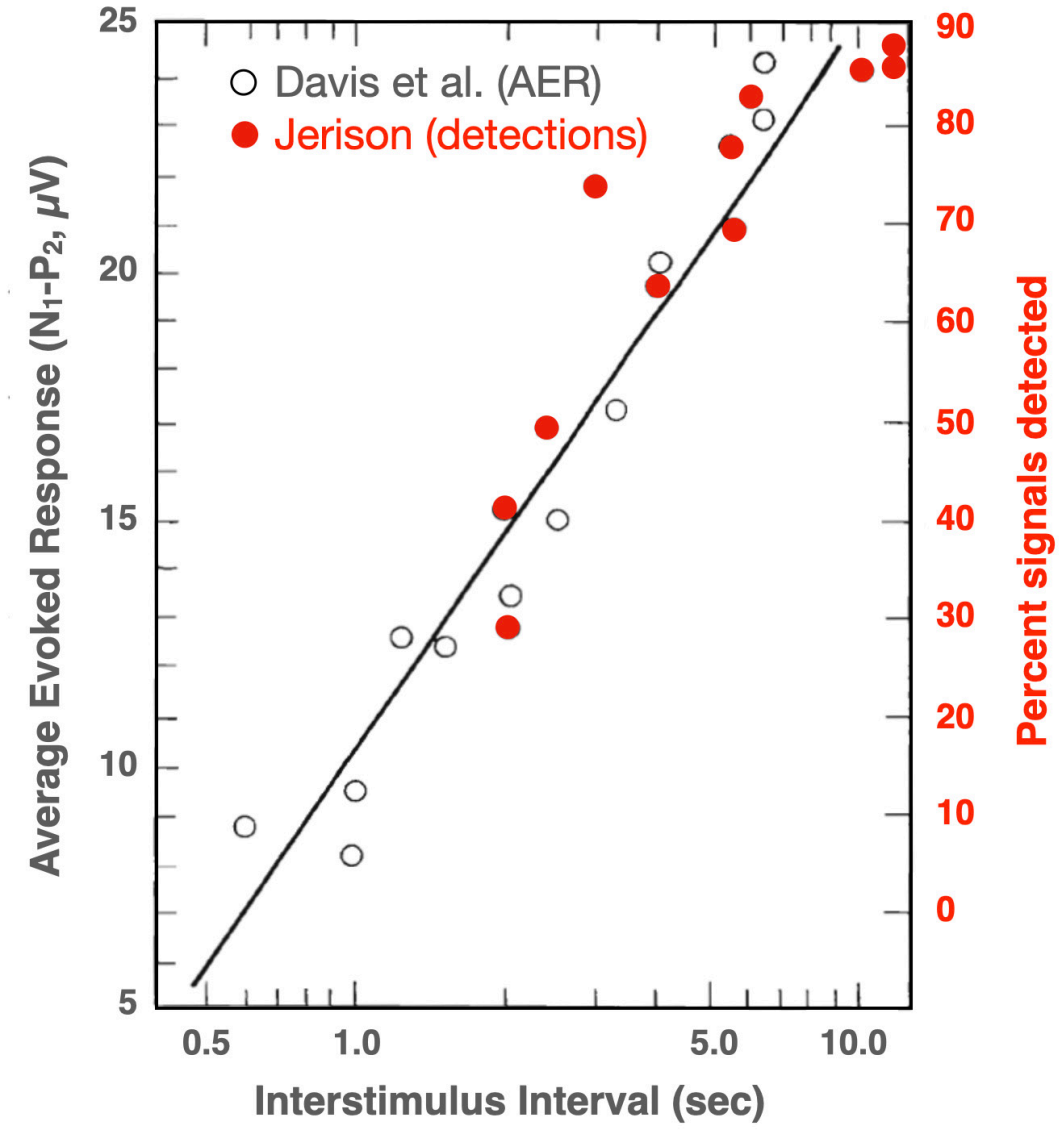
Effects of inter stimulus interval (on a log scale) on probability of detection from near the end of a visual vigilance task and average evoked responses to auditory stimuli (redrawn and corrected from Posner, 1975, who based his figure on Jerison, 1970, Figure 2).

Two types of vigilance tasks are contrasted, both of which show a reduction in target detections with time on task: Tasks with relatively high vs. relatively low rates of stimulation (principally non-targets). Posner notes that when processing a relatively rapid stream of items, the decline in performance is similar whether the target events in the sequence are relatively frequent or infrequent. In line with Mackworth's (1968b, 1969) habituation theory, Posner notes that there may be a relation between the decline in performance and reductions in the brain's responses to successive stimuli, and he reproduces a figure from Jerison (1970) to support this conjecture. To illustrate this relation, Jerison (1970, Figure 2) plotted the probability of detection toward the end of an 80-min vigil from his own vigilance experiments along with electrophysiological data (average evoked responses) from Davis et al. (1966), treating both as a function of the interval between stimuli. In reproducing this figure, Posner made a significant graphical error: neglecting to differentiate the data from the two different experiments. In Figure 4, this error has been repaired.

Despite the acknowledged looseness of the relation illustrated here, Posner sees it as consistent with the idea that at least two mechanisms might be involved in the vigilance decrement: In line with Broadbent's suggestion [(a) above], when the overall event rate is high the vigilance decrement manifests as a decrease in sensitivity (d′). In contrast, when the event rate is low, there is no decrease in sensitivity, and the decline in detections is due to a conservative shift in the criterion. Posner (1975) speculates that

high event tasks involve a pathway effect which specifically reduces the efficiency of processing for signals resembling the events being repeated, while low event rate tasks mainly involve a reduction in general alertness.

(p. 452)

In 1976, a second symposium on vigilance was held in St. Vincent, Italy. This one was co-sponsored by NATO and by the U.S. Office of Naval Research. The proceedings were published the next year in *Vigilance: Theory, Operational Performance, and Physiological Correlates*, edited by R. Mackie. Most of the 35 chapters were empirical with an emphasis on long, continued performance (often driving); sleep deprivation; physiological correlates; and time of day. Probably the most influential paper was titled “A Taxonomic Analysis of Vigilance Performance” by Parasuraman and Davies (see the following discussion for a brief summary by Swets).

In his introduction to the proceedings, Mackie (1977) advocates an eclectic approach to theory, a theme (anticipated by Broadbent, 1971, p. 11) that was later endorsed by Warm (1977):

The flurry of interest in vigilance research, spanning a period of nearly 30 years since Mackworth's early work, has led to a corresponding emphasis on the development of various theories of vigilance which now include (at least) activation or arousal theory, expectancy theory, signal detection theory, filter theory, observing response theory, inhibition theory, and reinforcement theory (Broadbent, 1971; Davies and Tune, 1969; Mackworth, 1970; Stroh, 1971). Early points of view reflected something of a competition among theories but more recent work is characterized by integrated positions that encompass contributions from several of these theoretical orientations (Jerison, 1967; Stroh, 1971).

(p. 1–2)

When summarizing his keynote chapter, Jerison (1977) identifies two important themes: first, that understanding vigilance and the decrement associated with long continued performance has important practical implications and, second, that vigilance is essentially “sustained” attention (see also Stroh, 1971, earlier book whose title, *Vigilance: The Problem of Sustained Attention*, asserts this equivalence):

I have argued the importance of our roots in the practical needs for good performance by watchkeepers, and have emphasized the fact that because of those roots we have developed a naturalistic environment for the study of sustained attention.

(p. 31–32)

After summarizing what Swets and Kristofferson had reported in their 1970 review, Swets (1977) goes on to review the 30 or so applications of SDT to vigilance since then, noting that about half of these new studies were industrial rather than laboratory-based. He summarizes that

the experiments of the '70s support the main result of the '60s: A vigilance decrement attributable to a decline in sensitivity cannot be taken for granted, and indeed, is not very likely across a set of experiments with typical display parameters. The consistent result of passing time is a progressively stricter decision criterion; according to the SDT analysis, this process frequently accounts for all of the decrement in the proportion of true detections, and almost always accounts for at least a part of that decrement. When a statistically significant decrement in d′ did occur, it was on the order of 20%.

(Swets, 1977, p. 710)

Regarding the industrial applications, he notes that

[s]trong support for the value of SDT to human factors specialists in industry is the testimony of Chapman and Sinclair (1975): “The practical value [of SOT] arises from the fact that it allows economic justification for the application of ergonomics to inspection, and [from] the relative ease with which recommendations for improvement can be derived (p. 241).

(Swets, 1977, p. 717)

In a footnote (added because in preparing his presentation, Swets was unaware of what Parasuraman and Davies (1977), would present in their paper “A Taxonomic Analysis of Vigilance Performance”), he says:

Raja Parasuraman's careful analysis in another paper in this volume shows that conditions leading to a sensitivity decrement can still be sharply defined, despite the existence now of auditory experiments showing a decrement in d′. A combination of two factors serves to isolate the tasks that lead to a decrement: an event interval of 2.5 seconds or less, and a signal that requires a “successive” comparison. The signal then consists of a change in a standard.

(Swets, 1977, p. 717)

**Figure 5:**
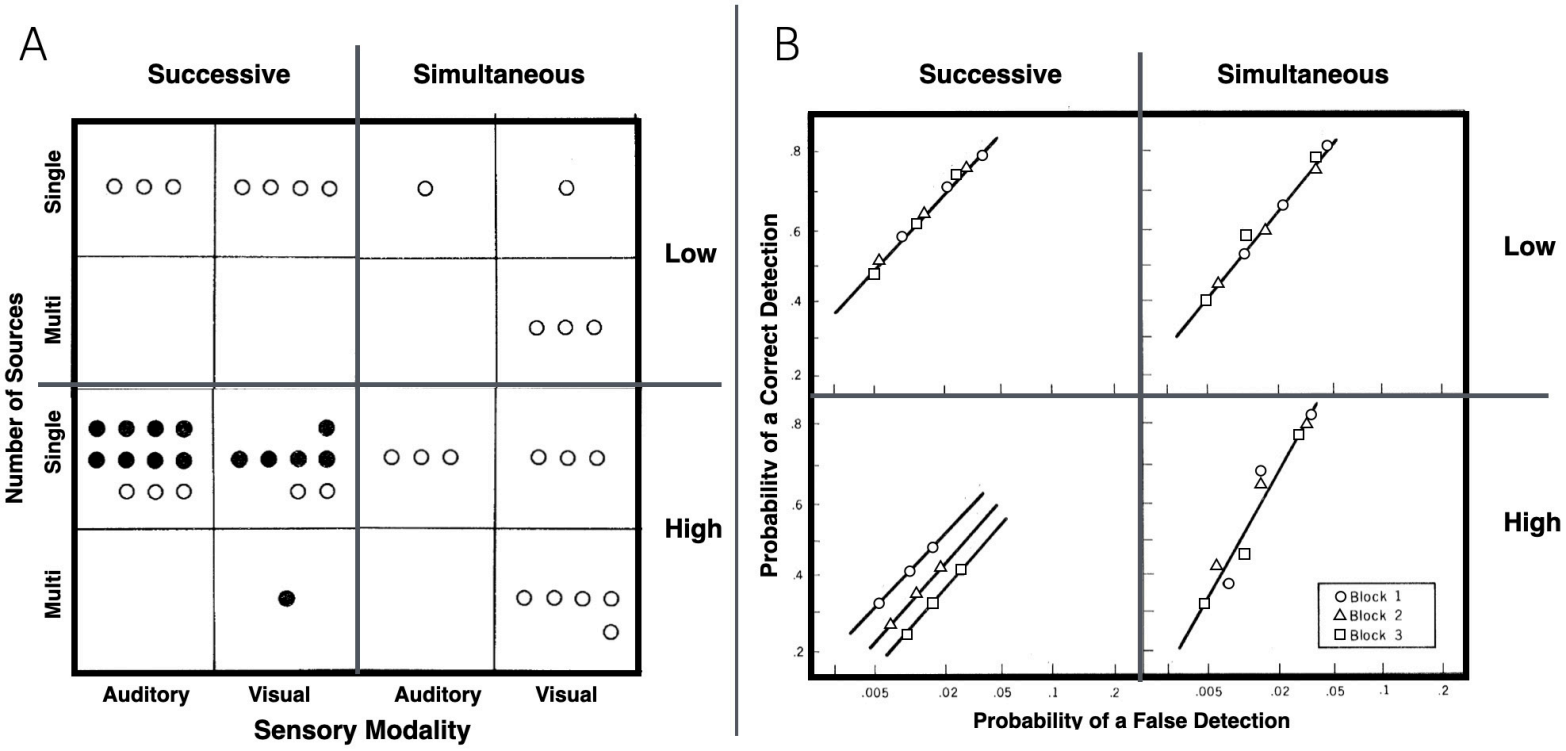
**(A)** Taxonomy of vigilance tasks, wherein each circle represents a task used in a study that reported a measure of sensitivity [d′, p(a), etc.] and filled circles denote the finding of a reliable decrement in sensitivity with time on task (redrawn from Parasuraman, 1979, Figure 2). **(B)** Results from four groups of participants performing either a successive or simultaneous auditory detection task with stimuli presented at either a low or high rate (redrawn from Parasuraman, 1979, Figure 1). After practice, participants performed the task for three blocks of 15 min each. Data plotted as circles, triangles, and squares represent the hit and false-alarm rates for blocks 1, 2, and 3, respectively.

Two years later, this theme was revisited by Parasuraman (1979). Here he reprises the Parasuraman and Davies (1977) analysis of the vigilance decrement literature within the aforementioned taxonomy with a focus on the question, When is the vigilance decrement characterized by a sensitivity decline? That analysis is presented in Figure 5A. After classifying tasks by sensory modality (vision or audition), source complexity (single or multiple sources), task type (simultaneous or successive), and event rate (high or low). Parasuraman concludes that sensitivity declines were confined to a combination of high event rate and a successive task. Sensory modality and source complexity were inferred to be unimportant. Importantly, while a sensitivity decline could be observed with this combination, it did not guarantee that one would be obtained.

Parasuraman's (1979) paper also included two experiments, a brief presentation of which will be useful for making the simultaneous/successive distinction clear. Experiment 1 used auditory stimuli presented either 15 (slow) or 30 (fast) times per minute. In the simultaneous task, each auditory event was a brief noise burst, and the target was an embedded 1,000-Hz tone. In the successive task, each auditory event was a 1,000-Hz tone, and the target was a small increase in loudness. To equate the conditional probability of a target across the two rates, targets were presented about once (for the slow rate) or twice (for the fast rate) per minute. For every event, the participant was required to make a four-category confidence rating, which allowed Parasuraman to infer d′ from ROC curves. As illustrated in Figure 5B, hit and false-alarm rates declined with time on task in all four conditions (almost certainly due to a conservative shift in the criterion), but in support of Parasuraman's analysis of the literature to that point, d′ declined only when the successive task was combined with a high event rate. Parasuraman's taxonomy is thus analytic for sensitivity, which only declines with time on task when the task is successive and the rate of stimulation is high, but it is not analytic for the conservative bias adjustment, which occurs robustly regardless of taxonomic classification.

Noting that the lack of a sensitivity decline in the simultaneous task could be due to either the absence of a “memory requirement”^4^ or the need to detect a stimulus in noise, a second experiment was conducted with three visual tasks. In the successive task, two circular light sources flashed 30 times per second. The target entailed a slightly lower intensity of both flashes. Two simultaneous tasks were used. In one task, the target was a slightly dimmer flash in one of the two signals. In the other, the target was a small circle appearing in the center of one of the signals. The participant simply pressed a key whenever a target was detected. Hence, d′ and beta would be computed from the hit and FA rates. As with the first experiment, hit rates declined in all three conditions, but d′ only declined with the successive discrimination task.

## 5 1981–1992

In 1995, See et al. published a meta-analytic review of the sensitivity decline in vigilance tasks. This review was jointly inspired by the advice in Swets and Kristofferson (1970) to determine which kinds of tasks yield sensitivity declines over time and which do not (see Section 4) and Parasuraman and Davies (1977; see also Parasuraman, 1979) earlier taxonomic effort to do so. The literature since about 1980 suggested to See et al. that another dimension might be important: Whether the relevant discrimination was sensory or cognitive. As described by See et al. (1995), “[s]ensory tasks are those in which the critical signals are predesignatcd changes in the physical characteristics of the stimuli (e.g., changes in auditory or visual intensity), whereas cognitive tasks are those that use symbolic or alphanumeric stimuli” (p. 232). As an example of a task with cognitive discrimination consider what was done by Nuechterlein et al. (1983), wherein every stimulus was a blurry digit and the target was the digit zero. See et al.'s meta-analysis included 42 studies (138 conditions) that were published in English between 1980 and 1992 for which the participants were adults and a measure of perceptual sensitivity was reported.

Where possible, for each of 138 conditions of the 42 studies, they computed effect sizes as follows:

[T]he effect size was computed as the difference between perceptual sensitivity scores during the first and last periods of a vigil, divided by the square root of the mean square error term for the time effect.

(See et al., 1995, p. 234)

**Figure 6:**
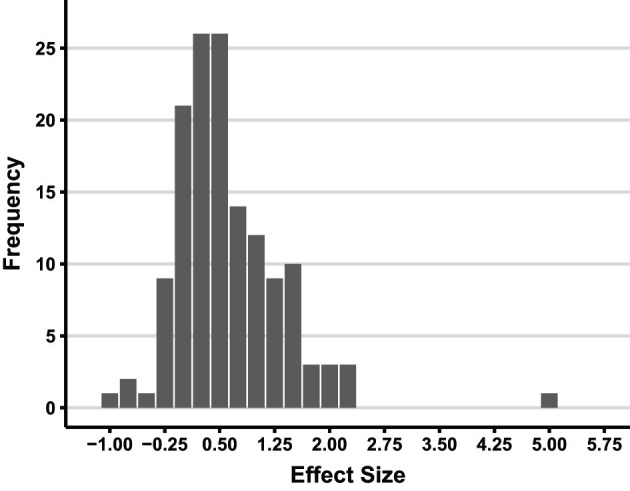
Distribution of effect sizes in the 138 conditions that explored vigilance decrements using signal detection theory between 1980 and 1992 (redrawn from Figure 2 in See et al., 1995).

Each condition was classified by task characteristics that had already been shown to affect vigilance or might be expected to do so. From the perspective of this review the most important findings are presented in Figure 6, Table 1. As can be seen in Figure 6, in the vast majority of the 138 conditions there was a positive effect size (decrement in sensitivity) with time-on-task. The average effect size as a function the variables in the authors' revised taxonomy (essentially simultaneous vs. successive tasks, event rate, and whether the stimuli were sensory or cognitive) is presented in Table 1.

**Table 1:** Number of conditions and effect size (decline in sensitivity during a vigil) as a function taxonomic classification (from See et al's, 1995, Table 6).

**Taxonomic classification**			**# of conditions**	**Effect size**
**Task**	**Event rate**	**Stimulus type**		
Simultaneous	Low	Sensory	7	0.91
Simultaneous	Low	Cognitive	1	0.00
Simultaneous	High	Sensory	30	0.74
Simultaneous	High	Cognitive	30	0.47
Successive	Low	Sensory	15	0.38
Successive	Low	Cognitive	3	0.78
Successive	High	Sensory	45	0.72
Successive	High	Cognitive	5	0.76
All			138	0.71

From these results See et al. (1995) draw this conclusion which is in strong contrast with that from research in the prior decades:

The current finding that the vigilance decrement may be accompanied by a reduction in perceptual sensitivity in the context of a wide variety of tasks and displays, however, implies that the sensitivity decrement may be much more prevalent than previously believed (Craig, 1987; Williges, 1969). The vigilance decrement appears to be associated with a genuine loss in the observer's ability to discriminate critical signals from non-signals not just in high-event-rate/successive-discrimination tasks but in a multitude of other situations as well.

(p. 242)

**Figure 7:**
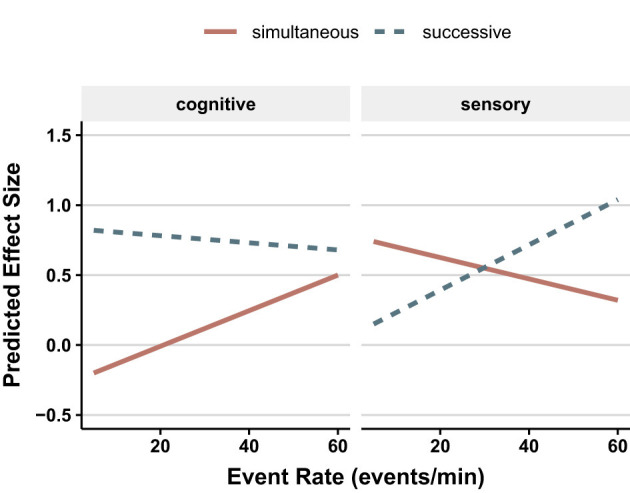
Effect size (of the vigilance decrement in d′) as a function of event rate and whether the task was sensory or cognitive. Upper panel is for simultaneous tasks; lower panel is for successive tasks (redrawn from Figure 3 in See et al., 1995).

This broad conclusion is somewhat constrained by the results from a multiple regression on condition-level effect sizes. This revealed significant effects of three task-related factors: type of discrimination (simultaneous vs. successive), type of stimulus (sensory or symbolic), and event rate that were qualified by a three-way interaction. As can be seen in Figure 7, with simultaneous discriminations using symbolic stimuli and sequential discriminations using sensory stimuli, there was a dramatic increase in the predicted effect size (decrement in sensitivity) as event rate increased, from near zero as the slowest rate to effect sizes of 0.5 (simultaneous/symbolic) and 1.0 (sequential/sensory) at the highest rates. In the remaining two conditions (simultaneous/sensory and sequential/symbolic), increasing event rate generated modest declines in predicted effect size.

## 6 1993–2010

Considerable theory development took place in next few decades (for some examples, see Section 8.1). Nevertheless, it is our view that this period is noteworthy because of the development and widespread use of two easy to administer tasks: The Psychomotor Vigilance Task (PVT, Dinges and Powell, 1985) and the Sustained Attention To Response Task (SART, Robertson et al., 1997).

First described in 1985, the *psychomotor vigilance task*, originally a microcomputer application, was specifically developed to explore the effect of sleep deprivation on long-continued performance. According to the paper's abstract,

[t]here is a need for brief, portable performance measures that are free of practice effects but that reliably show the impact of sleep loss on performance during sustained work. Reaction time (RT) tasks hold considerable promise in meeting this need, if the extensive number of responses they typically yield can be processed in ways that quickly provide the essential analyses.

(Dinges and Powell, 1985, p. 652)

**Figure 8:**
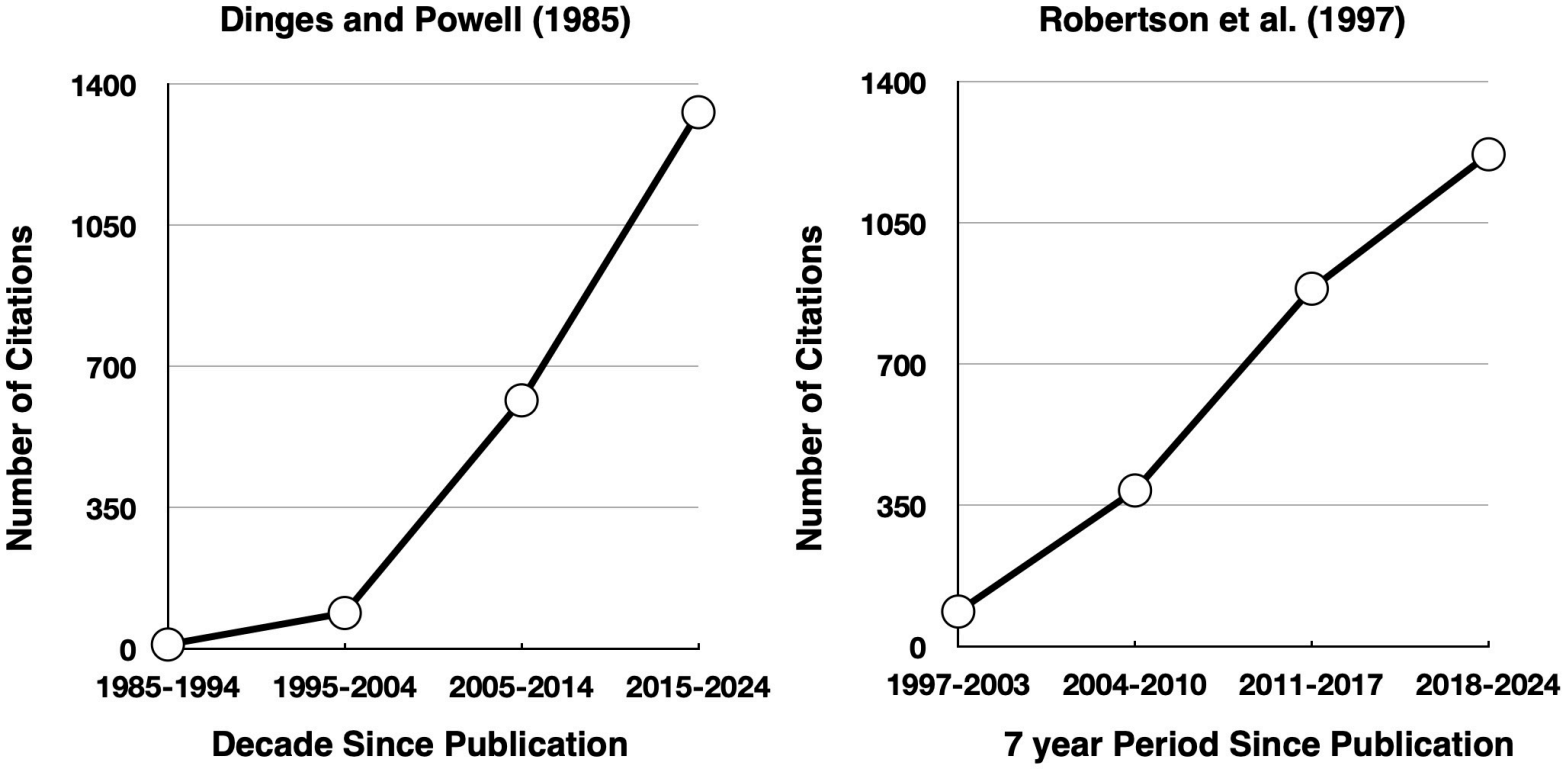
Number of times Dinges and Powell (1985) and Robertson et al. (1997) have been cited since their publication (from Google Scholar, December 2024).

Due to its simplicity, employing a single stimulus (the timer) and a single, simple outcome measure (RT), the PVT has almost no learning curve and allows for collecting upward of several hundred trials within a short testing session (typically 10 min or less). Researchers focus on various features of the RT distribution, with typical measures being the mean RT, the standard deviation of RT, the mean 10% slowest RT, and the proportion of “lapsed” responses, defined as RTs exceeding some threshold (conventionally, % of RTs ≥ 500 ms). The PVT has proven to be remarkably sensitive to sleep loss, with moderate-to-large effect sizes (Cohen's *d*) having been observed in response to partial (~4 h), and total sleep loss, even in PVT administrations as short as 3 min (Basner et al., 2011; Lim and Dinges, 2010). As illustrated in Figure 8 (left panel), this task gradually garnered immense interest and popularity. Indeed, of the 1,129 documents wherein “psychomotor vigilance task” was found in the title, abstract, or keywords, 885 of these were published in the last decade (from Scopus, December 2024). Although the great majority of these papers are about some aspect of sleep (e.g., deprivation, naps, etc.); PVT has recently been recast as a measure of a subtype of vigilance, referred to as “arousal vigilance” (Luna et al., 2018, and see Section 7), as it places no demands on response selection and simply requires the user to react quickly to a sudden stimulus onset.

As suggested by their title, “‘Oops!': Performance Correlates of Everyday Attentional Failures in Traumatic Brain Injured and Normal Subjects,” Robertson et al. (1997) were interested in what Broadbent et al. (1982) referred to as cognitive failures, particularly as a function of brain damage. The SART is a go/no-go paradigm in which a stream of individual digits (0–9) is presented, each separated by a fixed time interval. Upon seeing each digit, the user is instructed to make a detection response, save for one digit (e.g., 3), which serves as the no-go signal for which withholding the response is required. Supporting the validity and usefulness of the SART, Robertson et al. found that SART errors:

1. Correlated significantly with Cognitive Failures Questionnaire scores (whether reported by self or a relative) in a group of normal participants,
2. Correlated significantly with the severity of brain damage (Glasgow Coma Scale) in patients with traumatic brain injury (TBI), and
3. Were more frequent in TBI patients than in controls.

As illustrated in Figure 8 (right panel), the SART has also garnered immense interest and has been widely used to explore neuropsychological patients, aging, mind-wandering, sleep loss, and the effectiveness of various interventions thought to improve attention. Given the SART's requirement to selectively behave (i.e., make or inhibit responses), responses can be categorized as hits, false alarms, and so on, which allows for computing SDT metrics (i.e., sensitivity, bias, etc.) in addition to traditional RT and accuracy-based analyses. Indeed Bedi et al. (2024) recently concluded that SART errors were associated with a liberal response bias but, importantly, suggested that the SART stimuli “are simply too easy to discriminate to be considered a good candidate for a vigilance or sustained attention task” (p. 2040).

## 7 2011–present

In this most recent period, we see two important developments regarding exploring vigilance and its decrement with time on task: The development of an Attention Network Test (the ANTI-Vea) that was specifically designed to include measures of vigilance and challenges to the empirical generalization that a decline in sensitivity is a robust contributor to the decline in target detection.

Vigilant monitoring for targets necessarily requires attending to whichever medium and/or modality through which they appear. With that in mind, whatever the vigilance decrement may be, it should not be contentious to assert that it possesses some relationship with attention. In the early 2000s, building on Posner and Petersen's review of the brain's attention systems, Posner and colleagues (Fan et al., 2002; Fossella et al., 2002; Posner et al., 2002) developed and began to exploit what has since been referred to as the Attention Network Test (or ANT), which generates scores reflecting the efficacy of threes aspects of attention: alertness, orienting, and executive control. Shortly thereafter, Callejas et al. (2004, 2005) developed a modified version they named the ANT for interactions (or ANT-I). It is noteworthy that when measuring alertness, the ANT and ANT-I are assessing its phasic form—the rapid changes in arousal and preparation that accompany a warning signal.

Beginning around 2011, Lupianez and colleagues (Roca et al., 2011; Luna et al., 2018) began modifications of the ANT-I to incorporate an effort to measure vigilance: First, the ANTI-V and later the ANTI-Vea. Herein we keep our focus on the ANTI-Vea, as Roca et al.'s ANTI-V is effectively embedded within it. Luna et al.'s ANTI-Vea is composed of three sub-tasks, each with its own aims. The primary subtask is the ANT-I (comprising ~50% of trials). The ANT-I assesses the functioning of Posner's three networks of attention via auditory warning signals (alertness), uninformative visual cues (exogenous orienting), and a flanker discrimination task (executive control). The remaining two sub-tasks, both assays of vigilant performance, are motivated by the authors' observation that

[a]ssessing vigilance can be quite complex when variables such as task demands, engagement, and time on task are took into account (Thomson et al., 2015). Besides, *vigilance might not be a unitary concept*. Whilst this process is frequently described as *the ability to detect critical events through long time periods* (Warm et al., 2008), there are several studies that conceive vigilance as *sustaining the tonic arousal level that is necessary to react quickly to stimuli from the environment* (see, for example, Basner et al., 2013).

(Luna et al., 2018, p. 77; emphasis ours)

Luna et al. (2018) conceive of vigilance as having two subtypes, framing the “ability to detect critical events” as “executive” vigilance, and “sustaining the tonic arousal level… to react quickly” as “arousal” vigilance, hence the name “ANTI-Ve[executive]a[arousal].” To provide a measure of “arousal” vigilance, on a subset of trials (~25%) the typical ANT-I display is suddenly replaced PVT-like timer, to which participants must quickly halt via a non-specific keypress (e.g., any key). As a measure of “executive” vigilance, the remaining ~ 25% of trials proceed like typical ANT-I trials, with the exception that the central target arrow is vertically displaced by a small amount from its usual position. Upon detecting such displacements, participants are instructed to forgo discriminating the direction of the arrow and instead signal (via keypress) having noticed the change. Luna et al.'s “Ve” subtask shares an important feature with the SART in the occasional requirement to refrain from making the usual response, but unlike the SART, participants discriminate not when to make a response but what response to make.

Performance trends in the ANTI-Vea's subtasks replicate those seen in the “traditional” vigilance tasks they, respectively, mimic. Across “Va” (e.g., PVT-akin) trials, increases are seen in block-wise RT mean and standard deviation and in the proportion of lapses (excessively delayed RTs). Across “Ve” trials, decreasing proportions of hits and FAs are committed over time, which manifests as a statistically significant conservative shift in response bias, with no similarly significant decline in sensitivity (using A', the non-parametric analog to d′). In a study of our own (Feltmate et al., 2020) wherein each participant contributed four full-length runs of a modified ANTI-Vea, we observed a statistically significant decline in sensitivity across time on task in addition to a conservative bias shift. The magnitude of the sensitivity decline we observed (~from 0.905 to 0.886) is quite similar to what was found in Luna et al.'s ANTI-Vea with similar vertical displacements (~0.926 to 0.905). Support for the significant decline in sensitivity reported by Feltmate et al. comes from a large-scale comparison of laboratory vs. online experiments using the ANTI-Vea by Luna et al. (2021) who found a highly significant decline in A'. This putative decline in sensitivity is qualified later (see footnote ^5^ in section 5)

Thomson et al. (2016) took a hard stance against sensitivity-driven explanations of the vigilance decrement:

We challenge this assertion [of a sensitivity decline] on two fronts: First, we contend on a theoretical level that the metrics employed to measure observer sensitivity in modern vigilance tasks… are inappropriate and largely uninterpretable… Second, we present the results of an experiment that demonstrates that shifts in response bias… over time can masquerade as a loss in sensitivity.

(p. 70)

The core of their argument goes as follows: Declines in sensitivity manifest as increases in false alarms or decreases in hits, whereas conservative bias shifts manifest as commensurate declines in both. However, “modern” (their word) vigilance tasks are too brief and/or too easy to elicit a sufficient number of false alarms to observe a commensurate decline. Consequently, if false alarms are already at floor, then conservative bias shifts would manifest as sensitivity declines because the false alarm rate cannot get any lower.^5^

Thomson et al. (2016) empirically supported their argument employing a condition that encouraged a higher than typical rate of false alarms with occasional non-targets (lures) more similar to the targets than the much more frequent non-targets. Participants were tasked with indicating when a presented word represented a four-legged creature. For one group, all non-targets were commonplace objects. Critically, for the other, a subset of non-targets was replaced with lure words representing non-four-legged creatures. An elegant feature of their design was that for the lure group, the probability of targets and lures was equated. Sensitivity declines and conservative bias shifts were observed for both groups but only when calculated from all responses (e.g., lumping responses to lures and non-words together). When analyzing responses to targets and lures in isolation, the shift in bias remained, but no sensitivity decline was observed. Thomson et al. concluded:

[W]e submit that the general notion that the vigilance decrement owes primarily to a decrease in sensitivity has little basis in empirical fact… from the results of our empirical demonstration, we submit that the observed decreases in sensitivity metrics observed in many standard vigilance tasks may actually reflect shifts in response bias… without the appropriate test, it is not possible to delineate the role (if any) of sensitivity loss to performance declines in vigilance tasks.

(p. 79)

It is important to note that this paper has not gone unchallenged. In their own words, Fraulini et al. (2017) “discuss the theoretical, methodological, and procedural problems associated with the arguments described in Thomson et al. (2016).” In agreement with Fraulini et al., we note that large swaths of the vigilance decrement literature (empirical and theoretical) are ignored by Thomson et al. From the way it is written, readers are likely to come away from Thomson et al. thinking that a sensitivity decline rarely, if ever, is a factor in the vigilance decrement. But careful consideration of the title reveals the important, bolded, qualifier (hedge): “A Critical Examination of the Evidence for Sensitivity Loss in Modern Vigilance Tasks.” Such tasks are not specifically defined but the adjective *abbreviated* is used to describe them, and the SART and PVT would almost certainly fall under the umbrella.

**Figure 9:**
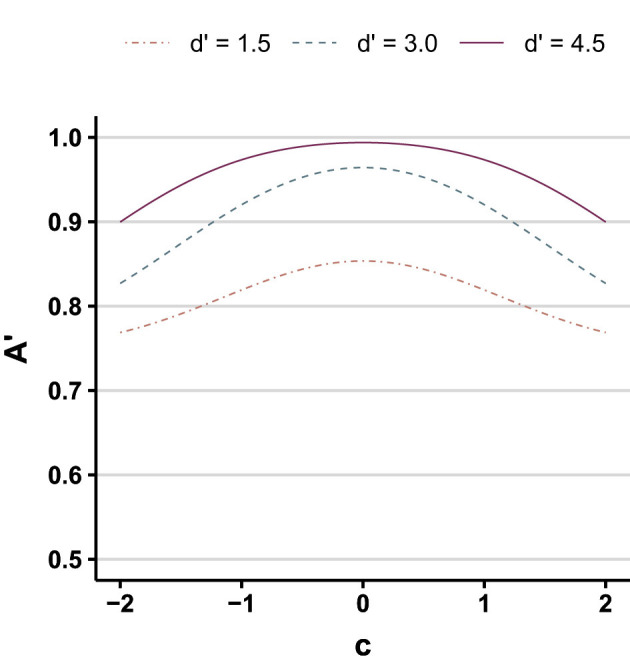
A' as a function of response bias (C) for three different values of true sensitivity (d′) (redrawn from McCarley et al., 2021, Figure 1).

Another wrinkle in this matter is the appropriateness of one's statistical methodology. McCarley et al. (2021) conducted a number of simulations to interrogate how sensitivity behaves, depending on the method used to compute it, when hit and false alarms approach their limits. As illustrated in Figure 9, their simulated results demonstrate that regardless of the true sensitivity (d′) bias shifts away from unbiased (either more conservative or more liberal) generate spurious declines in observed A'. The other sensitivity metric they explored, Az (a log-linear method wherein counts of hits/misses/etc. are incremented by 0.5; Hautus, 1995), was found to be resilient to floor effects in FAs when computed traditionally and when estimated via hierarchical Bayesian regression (wherein priors and partial pooling temper the influence of extreme values).

In a subsequent empirical paper, McCarley and Yamani (2021) stepped away from traditional SDT-based analyses by fitting participant responses to psychometric curves as a function of signal strength. By comparing curves generated from early vs. late performance, highly credible (in the Bayesian sense) evidence was found in favor of a sensitivity decline, a conservative drift in bias, and an increase in lapse rate (failures to respond). It is noteworthy that when Román-Caballero et al. (2022) applied this methodology to the data from Luna et al.'s (2021) large-scale study, the evidence for a sensitivity decline was replaced by evidence for lapses.

## 8 Epilog

Our exposure to and review of the vigilance decrement literature offers several themes for us to end with. First, although we have alluded to many explanations of the vigilance decrement, we have not presented any of them in any detail. Second, the vigilance decrement literature is vast relative to what we have presented, and while many important topics have been covered, there is one exciting development we would like to highlight: explorations of animal vigilance. Third, although this literature was initiated because of the practical importance of ameliorating the vigilance decrement in many real-world situations, in the field's efforts to test specific theories and try to understand the nature of the decrement, a degree of unreality characterizes many of these efforts. Finally, based on our inferences from reviewing the first 75 years of research on the vigilance decrement, we offer some recommendations.

### 8.1 Explanations for the vigilance decrement

Many explanations, often referred to as theories, have been offered for the vigilance decrement. Our purpose here is not to review all the proposals that have been advanced to explain the vigilance decrement—that would be another (somewhat daunting) project but rather to mention a few to give the reader a feel for the range of explanations and their historical contexts. Indeed, it is perhaps unsurprising that the proposed theories were often rooted in ideas that, at the time of their proposal, were either dominant or emergent in contemporary science.

Mackworth's (1948) proposal for the decrement, emphasizing *inhibition and extinction*, was embedded in contemporary ideas from the animal learning literature. Broadbent's (1958) proposal of a *filter theory* of attention provided a framework for thinking about the vigilance decrement—the filter, along with the target presented, is shifted away from the pathway. Whether this is regarded as an explanation or a description (e.g., what would cause it to shift away?), the idea was later incorporated into Jerison and Pickett's (1963) emphasis on “the observer's decisions about whether or not to attend to the display” (see Section 3). Jerison (1977) would later assert that “the vigilance task can be considered to provide a fundamental paradigm for defining sustained attention as a behavioral category” (p. 29), which he imagined as distinct from selective attention, the core of Broadbent's seminal theory. We do not see this as a distinction: What is meant by sustaining attention is selectively attending to an input pathway (or internal line of thought) for an extended period. Mackworth's proposal (Mackworth, 1968b, 1969), emphasizing *arousal and habituation*, was stimulated by Hebb's (1955) ideas about arousal and by contemporary research (Haider et al., 1964) and ideas (Sokolov, 1963) about habituation.

Today, the neuroscientific underpinnings of these phenomena are much better understood than they were over 50 years ago. Whereas, a review of this vast literature is beyond the scope of the present review, we offer some pointers to reviews for the interested reader. Placing vigilance into a general taxonomy of the brain's attention systems, the review by Posner and Petersen (1990) is a good starting place. These authors proposed that when “one needs to suspend activity while waiting for low probability events,” a right lateral midfrontal vigilance network is involved. This proposal was later validated and extended in a meta-analytic review of neuroimaging studies (with some linkage to lesion data) by Langner and Eickhoff (2013):

Integrating the results of both meta-analysis and review, a set of mainly right-lateralized brain regions was identified that may form the core network subserving vigilant attention in humans…

(p. 870)

After linking vigilance (sustained attention) to what is known about the sleep-wakefulness cycle, Oken et al. (2006) focus on physiological measures, primarily for assessing levels of alertness. A novel theory of the locus coeruleus-norepinephrine system that includes but extends its functions beyond simply arousal was presented by Aston-Jones and Cohen (2005). A general overview of the cognitive neuroscience of vigilance was provided by Sarter et al. (2001),

We have already covered the application of *SDT* for exploring vigilance behavior. This certainly is a theory, but it is not a theory of vigilance or its decrement with time on task. SDT provides a description of the decrease in target detections, characterizing it as a decline in sensitivity or an increase in the criterion for responding (or both). The application of SDT to the vigilance situation has revealed that there is often an increase in the criterion for responding with time on task. But it offers no explanation for why this shift might occur. Building on Deese's (1955) *expectancy* proposal, Craig (1978); Craig (1987) and Craig and Colquhoun (1977) provided one such explanation based on *probability matching*. Deese (1955) had suggested that the observer is “a detecting instrument that is continuously performing a kind of averaging of previous input in order to extrapolate the results to future behavior of the search field” (p. 364). Probability matching is the idea/finding that a well-trained and informed observer's rate of responding will match the rate of signal (target) presentations. As noted by Craig (1978):

Craig and Colquhoun (1977) recently reported that the behavior of subjects in an inspection study closely resembled that of naive observers in the psychophysical studies and that a substantial within-session decline in correct detections could reasonably be accounted for as an adaptive downward shift towards 1.00 in the R:S ratio.

(p. 442.)

Craig's (1978) analysis of the results of 30 published studies generated strong support for the notion that participants' initial overestimation of the probability of a target (and hence R:S ratio >1.0 and its reduction via probability matching is a major contributor to the increase in β that is associated with the frequently observed decline, with time on task, in target detections.

Many more recent proposals can be viewed as related in one way or another to concepts stimulated by the influential works of Baddeley and Hitch (1974), Kahneman (1973), and Shallice (1972; later, Norman and Shallice, 1986), which placed great emphasis on resources and executive control. To provide a few examples: *resource theory* (Warm et al. (2008), *resource depletion* (Warm et al., 2008; Wickens, 1980), *resource control* (Thomson et al., 2015), *mindlessness* (Manly et al., 1999), *mindwandering*^6^ (Cunningham et al., 2000), *failure of goal maintenance* (Ariga and Lleras, 2011) and *strategic resting* (Helton et al., 2011).

Proponents of each explanation for the vigilance decrement typically perform a hypothetic-deductive test of the sort alluded to earlier, often using the results to support their preferred theory while contesting another. The literature is replete with examples of factors that can accelerate the decrement (e.g., sleep loss and alcohol) or retard, if not eliminate, it (e.g., knowledge of results, stimulants, and interruptions). We are in complete agreement with Broadbent and others (see Section 4) that there will not be one single explanation for the vigilance decrement. There is an element of truth to each one, and they will often be operating simultaneously and to different degrees in different situations. Indeed, Warm's (1977) assessment still seems apt:

[T]he various theoretical models have focused upon somewhat different aspects of the vigilance problem and that they are not mutually exclusive. Most of the models can account for some but not all of the data and each invites criticism on several grounds.

(Warm, 1977, p. 623)

### 8.2 Vigilance in non-human species

In the preface to his edited volume, *Canine Ergonomics*, Helton (2009) relays this story from when he was a graduate student in the laboratory of William Dember and Joel Warm:

One day I was reading a paper about explosive detector dogs. The paper noted that dogs could search effectively only for 45 to 60 minutes, and then needed to rest. This sounded very familiar. The most ubiquitous finding in the sustained attention literature is the inability of humans to sustain their attention over long periods.

(p. x)

We are not sure if this putative similarity between humans and dogs has been empirically validates, but after demonstrating a vigilance decrement in 2-year-old rats, Grottick and Higgins (2002) infer “similar performance characteristics can be observed in both human and rat” (p. 33). Then they demonstrated that administration of psychostimulants (nicotine, amphetamine or caffeine) reversed the performance decrement.

**Figure 10:**
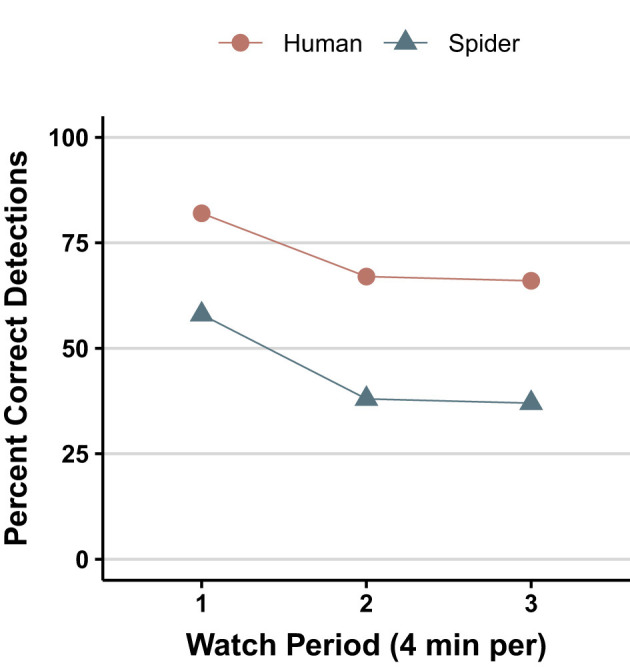
Response decrement with time on task in humans and jumping spiders (redrawn from Melrose et al. (2019, Figure 5).

An even more striking similarity (see Figure 10) was recently discovered by Melrose et al. (2019) in their comparison of humans and spiders. In a follow-up, Humphrey et al. (2019) demonstrated that administering caffeine to the spiders reduced the decrement. From this finding, the authors speculate “that, in at least in some invertebrates, CNS modulation of the vigilance decrement is likely” (p. 551).

Not all animals seem to behave like humans. Ridgway et al. (2009) explored target detection by dolphins over very long periods. They found

that dolphins were able to maintain cognitive ability as well as to detect goal tone stimuli at intervals during long vigilance trials to at least 120 continuous hours. [and suggested that] The ability of the dolphin to obtain sufficient sleep by uni-hemispheric means during intervals when a behavioral or vocal response is not required, most probably explains the dolphin's vigilance capability.

(p. 1,527)

### 8.3 Experimental control vs. ecological validity

Throughout the ~75-year history of research on the vigilance decrement, we see a tension between a desire to explore how vigilance operates in the real world and bring it under control in the laboratory to try to understand its nature. This tension between ecological validity and experimental control is a conundrum that regularly faces psychological scientists. This challenge was described by Klein and Hilchey (2010) as the psychological scientist “must ...navigate between uncontrol and unreality.” For example, in the effort to guarantee confidence in their measure of sensitivity, researchers developed tasks with sufficiently high false-alarm rates or required their participants to provide a confidence rating after each response. These two methodological procedures do improve the researchers' control, but they are in conflict with the real-world situations that stimulated the original research.

This tension has been regularly recognized by vigilance researchers. Indeed, as described in his introduction to the aforementioned 1976 NATO co-sponsored symposium, Mackie (1977) noted that “one focus of the symposium was on the operational relevance of vigilance research” (p. 4) and he believed that the symposium achieved a reasonable balance between applied and basic “forces.” In Jerison's (1977) presentation at this symposium, he relates the following interaction he had with the legendary Don Norman:

At that time the theory of signal detectability was everyone's hoped for panacea for resolving the problems of psychology. Vigilance was no exception. However, there was one major difficulty in applying the theory to the vigilance problem. One needed a reasonable number of false alarms to determine values for the parameters of the theory, and it is notorious that most observers in vigilance studies make few or no false alarms. Norman's view was that the experimental situation should somehow be modified to induce observers to emit false alarms. Now that may very well be good advice if signal detection theory provides the correct theory for vigilance. My view, on the contrary, was that the correct theory was unknown whereas the experimental paradigm (which 1 took as Norman Mackworth's clock test) really stated the basic problem. Furthermore, the evidence that few false alarms were made was itself suggestive that the theory of signal detectability might not be correct for the vigilance problem though it might be required as part of a complete theory. To manipulate the false alarm rate deliberately might, in fact, remove those elements from the vigilance task that make the task a suitable vehicle for studying sustained attention.

(p. 31)

Klein and Hilchey likened the need to avoid unreality and uncontrol to that of Odysseus in Homer's *The Odyssey*. Odysseus (and in other myths other sailors) was tasked to navigate between two sea monsters, the multi-headed, cliff-dwelling Scylla and the whirlpool-creating Charybdis. Klein and Hilchey advised psychological scientists to *avoid the hazard you most fear; risking the dangers of the other; hope for the best; navigate carefully, respecting (compromising with) both hazards… [and importantly] send out several ships with different navigational strategies*. Their message was that an individual researcher can endure the hazard of unreality or uncontrol so long as the field uses a diversity of strategies. Methodological hegemony, then is the biggest risk.

### 8.4 Some inferences and recommendations

The vigilance decrement is important in many real-world situations; therefore, so too is understanding its nature. The application of SDT has been valuable for this purpose, but it must be accompanied by caution when generalizing to real-world situations that simply are not well-matched by psychophysical paradigms. It is now clear that a measured decline in sensitivity can be an artifact generated when FA rates are at floor or by periods of lapsed responding or guessing. Nevertheless, the kind of whole-scale rejection of a sensitivity decline in all vigilance situations (implied by Thomson et al., 2016) is certainly unwarranted. The kind of taxonomic work encouraged by Swets and Kristofferson (1970) and later employed by Parasuraman (1979; Parasuraman and Davies, 1977) and See et al. (1995) ought to be revisited using methods that avoid the possibility of artifactual declines in measured sensitivity.

## Footnotes

1 Whereas Broadbent and Gregory may not have come up with a satisfactory explanation for the discrepancy between their findings and those of Mackworth and Taylor, their suggestion that “situations involving difficult sensory adjustments may actually show a drop in the sensitivity” was later supported by See et al's (1995) meta-analysis, which demonstrated that “[a]s a task becomes more demanding, the overall level of sensitivity associated with the task is reduced and the decrement increases in magnitude” (p. 243).

2 From a scholastic perspective, we would be remiss if we did not mention the two books about vigilance published by Jane Mackworkth in the Penguin Science of Behavior series at the beginning of this period. The first one (Mackworth, 1969) expanded on her ideas (Mackworth, 1968b) about habituation and arousal, while the second one (Mackworth, 1970) focused on attention and began with some coverage of SDT.

3 ROC curves are a psychophysical tool in SDT for obtaining a reliable measure of sensitivity (d′). They can be generated by manipulating the observer's criterion, thereby generating multiple values of hit and FA rates for the same detection situation. An alternative and more efficient method is to obtain, along with each target present and target absent decision the observer's confidence in their decision. Either way, the multiple data points are fit by a best fitting function and d′ can be computed from this function. The interested reader is referred to (Wixted 2020) for an engaging history of signal detection theory wherein this tool is well illustrated.

4 Parasuraman uses this term to describe the successive task because to detect the difference between the target and the standard memory of the previous stimulus is required. We are not sure that this is the critical difference. Consider Posner's point that the contribution of habituation in a vigilance task depends on the similarity between the target and non-target events. This would be high for 1000-Hz tone that only varies in intensity, whereas for with noise bursts that contain a 1,000-Hz tone and those that do not, habituation might not even affect processing of the tone.

5 Support for this suggestion can be found in the discussion section of (Luna et al. 2021), wherein they demonstrated that the aforementioned sensitivity decline was not observed in participants who began the test with a relatively high FA rate; it was confined to those participants whose FA rate was close to the floor (see also McCarley et al., 2021, p. 24).

6 This possibility (with a less exciting nomenclature) was anticipated by (Broadbent 1958, p. 108), who wrote: “We have come to the conclusion that after prolonged observation of one source of information, a man will show brief intervals in which he takes in information from other sources. His attention will wander.”
